# Precision detection of crop diseases based on improved YOLOv5 model

**DOI:** 10.3389/fpls.2022.1066835

**Published:** 2023-01-09

**Authors:** Yun Zhao, Yuan Yang, Xing Xu, Cheng Sun

**Affiliations:** School of Information and Electronic Engineering, Zhejiang University of Science and Technology, Hangzhou, China

**Keywords:** diseases detection, YOLOv5S, CSP structure, CAM structure, loss function

## Abstract

Accurate identification of crop diseases can effectively improve crop yield. Most current crop diseases present small targets, dense numbers, occlusions and similar appearance of different diseases, and the current target detection algorithms are not effective in identifying similar crop diseases. Therefore, in this paper, an improved model based on YOLOv5s was proposed to improve the detection of crop diseases. First, the CSP structure of the original model in the feature fusion stage was improved, and a lightweight structure was used in the improved CSP structure to reduce the model parameters, while the feature information of different layers was extracted in the form of multiple branches. A structure named CAM was proposed, which can extract global and local features of each network layer separately, and the CAM structure can better fuse semantic and scale inconsistent features to enhance the extraction of global information of the network. In order to increase the number of positive samples in the model training process, one more grid was added to the original model with three grids to predict the target, and the formula for the prediction frame centroid offset was modified to obtain the better prediction frame centroid offset when the target centroid falled on the special point of the grid. To solve the problem of the prediction frame being scaled incorrectly during model training, an improved DIoU loss function was used to replace the GIoU loss function used in the original YOLOv5s. Finally, the improved model was trained using transfer learning, the results showed that the improved model had the best mean average precision (mAP) performance compared to the Faster R-CNN, SSD, YOLOv3, YOLOv4, YOLOv4-tiny, and YOLOv5s models, and the mAP, F1 score, and recall of the improved model were 95.92%, 0.91, and 87.89%, respectively. Compared with YOLOv5s, they improved by 4.58%, 5%, and 4.78%, respectively. The detection speed of the improved model was 40.01 FPS, which can meet the requirement of real-time detection. The results showed that the improved model outperformed the original model in several aspects, had stronger robustness and higher accuracy, and can provide better detection for crop diseases.

## 1 Introduction

Crop diseases have always been a major concern for farmers, and they can seriously affect the yield of crops. For crop disease and pest problems, manual methods were used in the past for pest removal, which have great limitations, such as being time-consuming and labor-intensive, as well as inaccurate grasp of the type, location and number of diseases at different times. Therefore, it is necessary to study a model that can detect crop diseases quickly and accurately. For the various diseases that exist in crops, scholars have been improving and innovating artificial intelligence techniques applied in agriculture, expecting to study an efficient and accurate model. In the field of computer vision includes several tasks such as image classification, target detection, instance segmentation, semantic segmentation, etc., and target detection is the most basic task among them. When convolutional neural networks were not yet developed, most target detection research used traditional algorithms, such as decision trees, Bayesian classification, Adaboost and support vector machines. Some scholars have compared the performance of both migration learning methods and deep feature plus SVM on 11 models, and the results showed that the latter had better results (Sethy et al., 2020). A combined fractional-order Zernike moments (FZM) and SVM approach was proposed to identify grape leaf diseases (Kaur et al., 2019). Two different methods based on traditional and deep learning were compared for extracting pepper pest and disease features and it was concluded that the deep learning based method has better performance (Ahmad Loti et al., 2021). A CNNs model called LeafNet was built using different sizes of feature extraction filters that were used to detect tea tree disease types and the average classification accuracy of this model was improved by 29.54% and 19.39% when compared with the SVM algorithm and MLP algorithm respectively (D.Pujari et al., 2016). Healthy and undesirable leaves of citrus were compared using the LIBS technique and classified using both quadratic discriminant analysis and SVM models in order to provide an effective nutritional evaluation method for citrus orchards (Sankaran et al., 2015).

After that, due to the rise of convolutional neural networks, target detection entered the period of using deep learning algorithms, where the development of target detection went through a process from second-order to first-order algorithms, first-order algorithms include SSD (Liu et al., 2016) and YOLO (Redmon et al., 2016; Redmon and Farhadi, 2017; Redmon and Farhadi, 2018; Bochkovskiy et al., 2020) family of algorithms, and second-order algorithms include R-CNN (Girshick et al., 2014), Fast R-CNN (Girshick, 2015), and Faster R-CNN (Ren et al., 2015) algorithms. From second-order algorithms to first-order algorithms, there are many scholars who have done various different studies. An improved Faster R-CNN method was proposed to detect four common diseases of tomato, and the improved method has improved the recognition accuracy by 2.71% over Faster R-CNN (Zhang et al., 2020). By combining the CBAM attention mechanism, HRNet network and ASPP structure to improve the R-CNN network, a detection algorithm was proposed to extract small target pests at different scales in citrus with an average recognition rate of 88.78% (Dai et al., 2021a). A fusion of FCM-KM and Faster R-CNN was proposed to detect rice diseases (Zhou et al., 2019). By comparing the Faster R-CNN models of eight different pre-trained networks to detect potato shoots, the experimental results showed that the average accuracy of the improved Faster R-CNN improved by 5.98% over the original Faster R-CNN (Xi et al., 2020). An algorithm called MFaster R-CNN was implemented by constructing a hybrid loss function using a central cost function and using four different pre-training structures, which can improve the detection of maize diseases in real environments (He et al., 2022). Using the Faster R-CNN algorithm to detect diseases associated with rice leaves, the detection accuracies of three diseases, rice blast, brown spot and hispa, were 98.09%, 98.85% and 99.17% (Bari et al., 2021). Compared with the second-order target detection algorithm, the first-order algorithm has higher detection accuracy and detection efficiency. Some scholars have provided technical support for robotic intelligent citrus picking by pruning the backbone network of YOLOv4 algorithm and proposing a two-way pyramidal network (Bi-PANet) (Zheng et al., 2021). An efficient network for detecting grape leaf pests was constructed by combining Inception structure, depth-separable convolution and dense connectivity structure, the accuracy of the model could reach 97.22% (Liu et al., 2020). Improved detection of tomato pests and diseases by improving the residual unit in YOLOv3 algorithm with 92.39% accuracy of the improved algorithm (Liu and Wang, 2020). An improved Faster DR-IACNN method for detecting common foliar diseases of grapes was proposed, which achieved 81.1% mAP and 15.01 FPS detection speed (Xie et al., 2020). An improved YOLOv4 method was proposed to detect plums of different maturity in orchards for their small shape and dense growth (Wang et al., 2022). An improved YOLO-Dense method (Wang and Liu, 2021b) and an improved YOLOv3-Tiny model (Wang et al., 2021) were proposed to detect tomato anomalies in complex natural environments. A multi-scale parallel algorithm MP-YOLOv3 was proposed to improve the detection of tomato gray mold based on the MobileNetv2-YOLOv3 model (Wang and Liu, 2021a), and the experimental results showed that the improved model has strong robustness in real natural environments. An algorithm based on super-resolution image enhancement (Zhu et al., 2021) and an algorithm combining Inception and an improved Softmax classifier are proposed to detect grape and apple diseases, respectively (Li et al., 2022). Enhancing feature extraction by incorporating DenseNet interlayer density in the YOLOv4 model (Gai et al., 2021). To improve the yield of carrots, a lightweight and improved YOLOv4 model was proposed, which uses a variety of lightweight structures to greatly reduce the number of parameters and the computational effort of the network (Ying et al., 2021). A class loss function based on AP-Loss (Average Precision loss) is proposed to deal with the positive and negative sample imbalance problem during training. The final experimental results show that the mAP of the improved algorithm is 97.13% (Chen et al., 2021). An improved YOLOv5 model was proposed to improve the detection of kiwi defects by adding a small target layer, introducing SE attention mechanism and CIoU loss function, and the mAP of the improved model was improved by nearly 9% compared with the original model (Yao et al., 2021). An improved YOLOv5 model incorporating the involution bottleneck module and the SE module was proposed (Chen et al., 2022), as well as a lightweight YOLOv5-CS model that improves the generalization capability of the model using image rotation coding to achieve accurate counting of citrus by deploying it into an AI edge system (Lyu et al., 2022).

As mentioned above, in the field of target detection, many scholars have proposed very good ideas and methods, which have been applied to agriculture with good results, but there is room for further improvement for certain agricultural disease problems, such as for the problem of small, dense and overlapping disease targets, many methods have high accuracy for disease detection but also high model computation, or the opposite of both. And in non-agricultural aspects also scholars have made research for small target datasets, such as one proposed an attention feature fusion structure to fuse semantic and scale inconsistent features (Dai et al., 2021b). Then a combination of MobileNetv2, YOLOv4 and attentional feature fusion structure has been proposed for the detection of underwater small targets and target aggregation to improve the accuracy by improving the attentional feature fusion structure to better fuse features of different scales (Zhang et al., 2021b). The detection accuracy of small aircraft and ship targets is improved by adding an additional detection layer and introducing union-non-maximum suppression (NMS) to the YOLOv5 model (Tan et al., 2021). In order to enhance the utilization of shallow convolutional features, a three-layer pyramidal network structure based on horizontal connection fusion is established, which can improve the detection of small targets (Lu et al., 2020). In this paper, we hope to propose a model with low number of parameters and high accuracy. Although the above methods are not applied in agriculture, they are also for some small and dense data sets, so we can get inspiration in them. In this paper, we consider extracting feature information of disease targets from different angles, obtaining different feature information from different structures of different branches, and improving the detection of disease targets by fusing feature structures containing different semantic In this paper, we consider extracting feature information of disease targets from different angles, obtaining different feature information through different structures of different branches, and improving the detection effect of targets by fusing feature structures containing different semantics information, and the improved model can achieve the expected effect through continuous comparison experiments.

## 2 Materials and methods

### 2.1 Materials

#### 2.1.1 Image and data accquisition

The images used in this paper were taken from the PlantVillage (Hughes and Salathé, 2015) public dataset. Five crops with eight disease types were taken from the PlantVillage dataset and manually annotated using the LableImg image annotation tool, and a total of 1319 images were annotated. In order to prevent the problem of overfitting and non-convergence due to too little training data, random image enhancement processes, including Gaussian blurring, horizontal flip, random rotation, and random brightness adjustment, were applied to the annotated 1319 images to expand the dataset to 4079 images. The expanded images were divided into the training set, validation set and test set, where the ratio of the training set to the test set is 9:1, and 10% of the training set is used as the validation set. The categories and numbers of diseases in the labeled dataset are shown in **Table 1**.

**Table 1:** Type and number of diseases in the labeled datasets.

Type	Number
Grape Black Measles	140
Grape Leaf Blight	180
Grape Black Rot	136
Peach Bacterial Spot	150
Potato Late Blight	316
Apple Black Rot	132
Apple Scab	114
Corn Northern Leaf Blight	151
Total	1319

#### 2.1.2 Image enhancement

**Figure 1:**
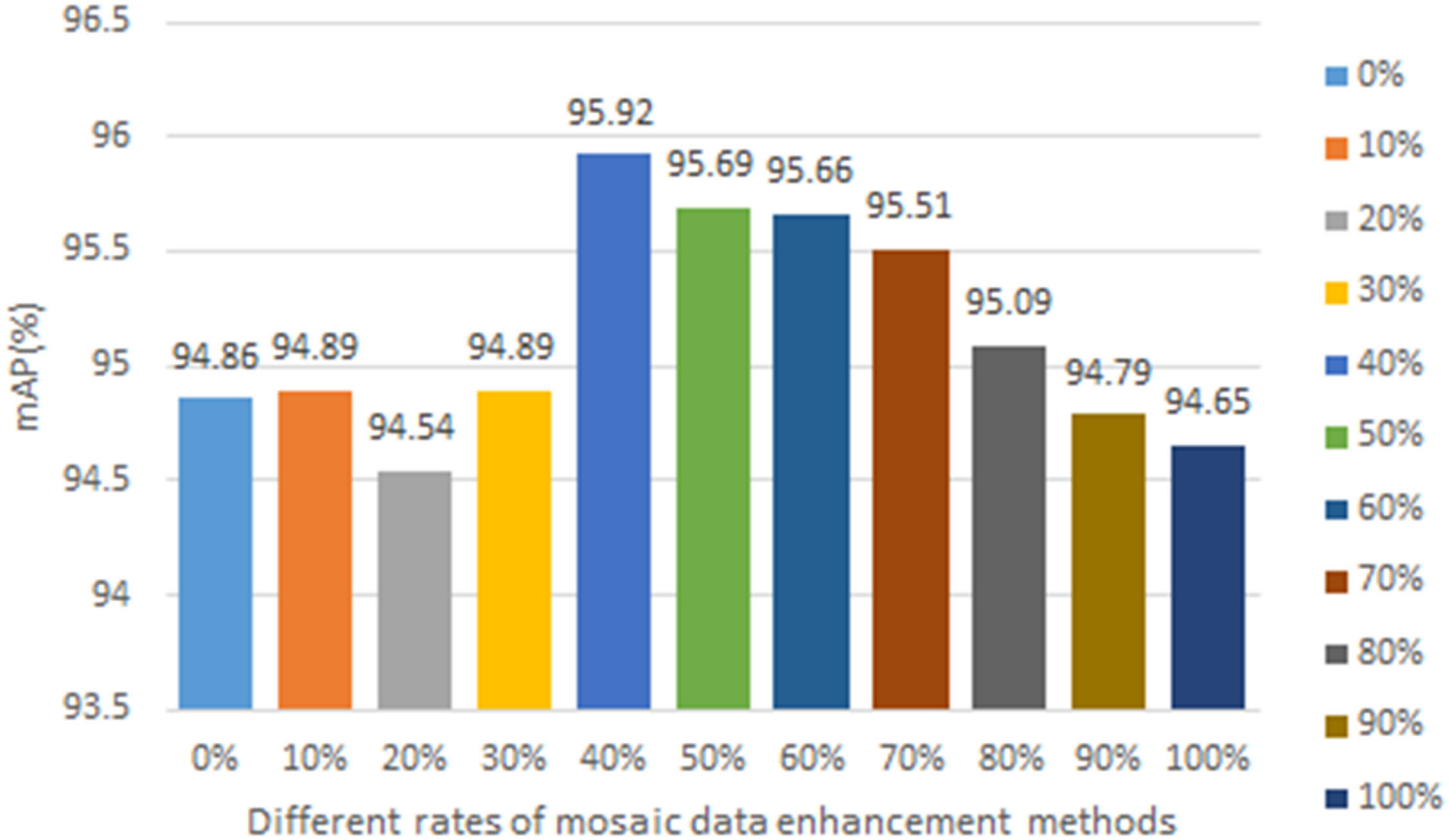
Different rates of mosaic data enhancement methods.

In this paper, we used two data enhancement methods in the training process, one is the common data enhancement method, such as image flipping, scaling, length and width distortion, and color gamut transformation, and the other is the mosaic data enhancement method. The mosaic data enhancement method is proposed in the YOLOv4 paper, and the main idea was to crop four images randomly and then stitch them onto one image as training data. Although the mosaic data augmentation method can expand the diversity of data samples and enhance the feature extraction ability of the network, it will be detached from the real distribution of natural images, so this paper used the mosaic data augmentation method in the first 40% of training rounds and the normal data augmentation method in the next 60% of rounds, so as to improve the training effect of the network. The mAP values obtained using different ratios of mosaic data augmentation methods for the improved model are shown in **Figure 1**. From the figure, it can be seen that the optimal results are obtained when using 40% of the mosaic data enhancement method.

### 2.2 Methods

#### 2.2.1 Improvement of the CSP structure

**Figure 2:**
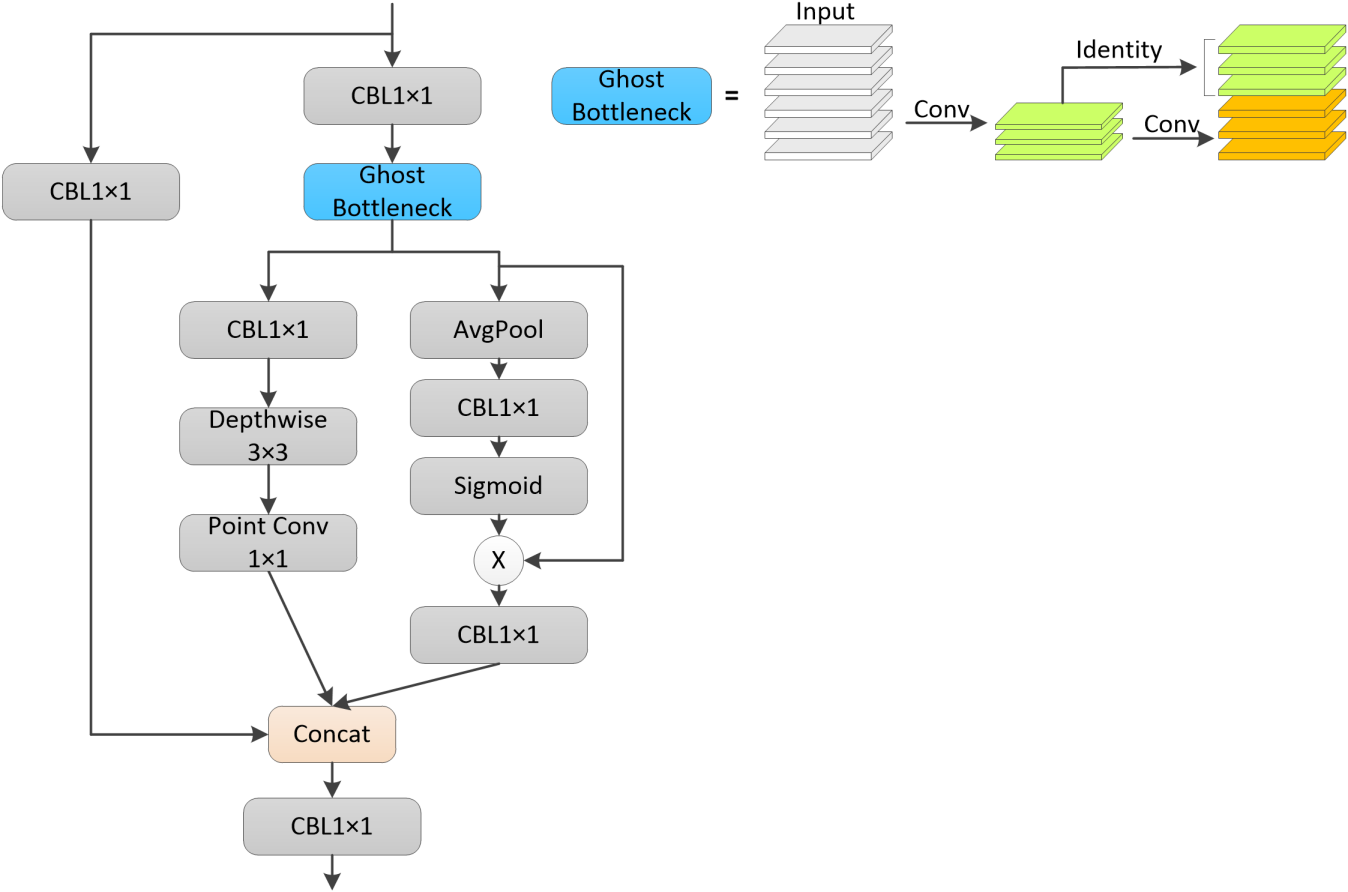
Improvement of the CSP Structure.

Compared with the YOLOv4 model, the YOLOv5 model used two different CSP structures in the backbone part and neck part. In this paper, the CSP structure of the neck part of the YOLOv5s model is improved, and the improved CSP structure is shown in **Figure 2**. The original CSP structure divides the feature mapping of the input layer into two parts, and then combines them after a series of convolution operations, which can reduce the computational effort and ensure the accuracy at the same time. In this paper, this design idea was retained in the improved CSP structure, and two lightweight structures were used to extract feature information. One is the Ghost module (Han et al., 2020), which not only generates more feature maps instead of normal convolution, but also has lower computational effort. The other is the inverted residual structure, which is proposed in the MobileNet (Howard et al., 2017; Sandler et al., 2018; Howard et al., 2019) family of networks and contains the main structures of deep convolution and point convolution. The modified CSP structure divided the upper layer input into two parts, each of which is passed through a convolutional layer to adjust the channel dimension, after which the output of one of the branches is passed through the Ghost module to replace the two normal convolutional layers in the original CSP structure, which is able to reduce the computational effort of the model while ensuring its accuracy, and then the output of this part was divided into two parts, one of which is passed through an inverse residual The structure of this part was divided into two parts, one was through a structure of inverted residuals, which first makes the number of input and output channels of deep convolution more by raising the dimensionality of one convolutional layer, so as to extract more information. The core of deep convolution is the number of input and output feature matrix channels is equal to the number of convolutional kernels, which can greatly reduce the model computation and the number of parameters. The other part was through a global average pooling layer to extract global information and enhance the ability of global information extraction. These two parts was able to extract different feature information from the input layer separately, avoiding the data redundancy caused by repeatedly extracting the same features. Finally, the outputs of these three parts were stitched into the channel dimension, and then the final number of output channels was adjusted by a convolution layer. The improved CSP structure was more complex than the original CSP structure, but the computational effort was not significantly increased because some lightweight structures were used, but the improved CSP structure can extract more feature information and enhance the detection effect of the model.

#### 2.2.2 The proposed CAM structure

**Figure 3:**
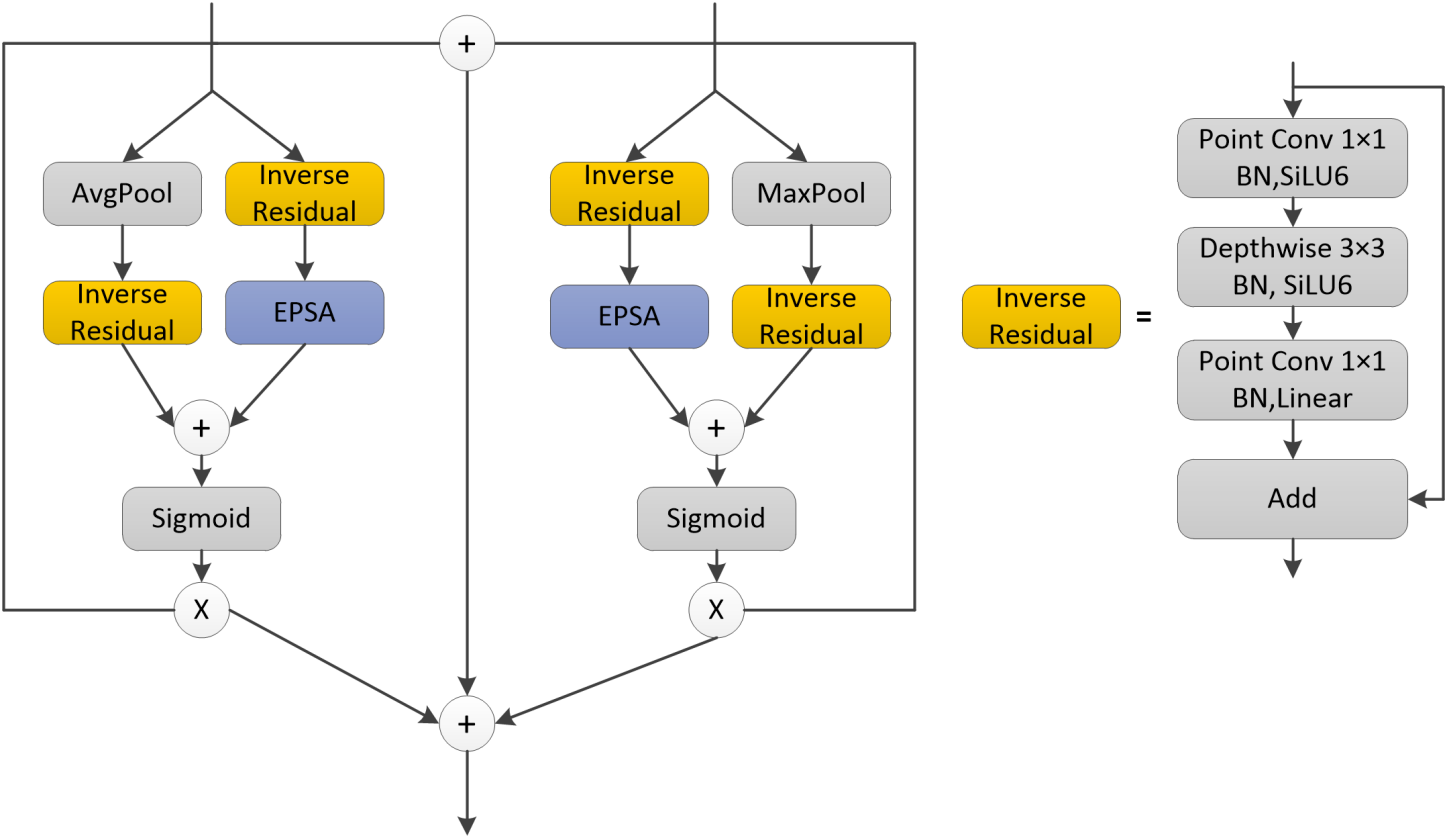
CAM structure.

This paper also proposed a module called CAM, which used a plug-and-play efficient multiscale channel attention mechanism that improves the channel attention mechanism, namely the EPSA module (Zhang et al., 2021a), and the structure of the CAM module is shown in **Figure 3**. The input of both deep and shallow networks was divided into two branches, where one branch of the deep network extracted the global features of the network through the global average pooling layer, and the other branch extracted the spatial information of the multi-scale feature map through the inverse residual structure and EPSA module. Finally, the two branches were fused for better feature fusion of different feature information. The shallow network’s input is divided into two branches, unlike the deep network, because one branch of the shallow network extracted the local features through the global maximum pooling layer, and the rest of the network was also enhanced by the inverse residual structure and EPSA module to extract the multi-scale feature maps. Global average pooling and global maximum pooling produce different effects in different layers. In the shallow network, the abstraction of the image is not yet very high, when the image contained more texture feature information, while as the network deepens, the resulting feature maps become more and more abstract, when the image contained more semantic and contextual information. Therefore, the input of the accepted shallow network in the CAM structure was passed through the global maximum pooling layer, through which some invalid information was removed to make the obtained feature maps more sensitive to detailed information. The input from the deeper network was accepted through a global average pooling layer to integrate global spatial information and obtain global contextual information. Secondly, the CAM module also fused the feature information of the two upper layer inputs, preserving the feature information of the original input and supplementing the feature information lost after the pooling operation. The CAM module can realize the feature fusion of global and local features, which can better fuse the features with inconsistent semantics and scales, thus enhancing the extraction capability of the global information of the network and improving the accuracy of the network model.

#### 2.2.3 Improvement of the bounding box prediction method

**Figure 4:**
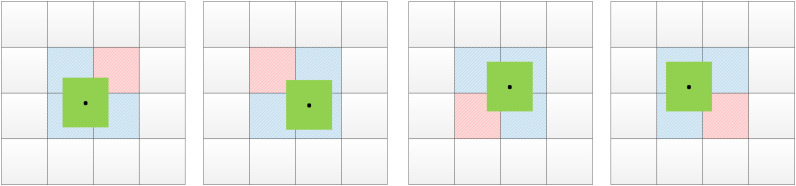
The prediction grid corresponding to the different positions of the real box.


In the YOLOv5s network model, the centroids of the preset anchor values all fall in the upper left corner of the prediction grid, and then are gradually shifted toward the target centroids by the formula. The real frame is mapped onto the feature map, and the target is predicted by the grid when the centroid of the real frame falls in a grid. The YOLOv5s network selects two additional grids adjacent to the grid in addition to the grid where the centroid of the real frame is located to predict the target, so as to increase the number of positive samples for model training. The improved YOLOv5s network added a neighboring grid to the original network to predict the target, and the prediction grid used when the center point fell at different locations is shown in **Figure 4**, where the green box is the real box, the black dot is the center point of the real box, the blue box is the prediction grid used in the original network, and the red box is an additional prediction grid added in this paper. The additional grid can further increase the number of positive samples and improve the accuracy of model predictions. The Formula of the original network for grid prediction offsets is:

(1) $$b_{x}\;=\;\left(2\times \sigma \left(t_{x}\right)-0.5\right)+\;c_{x}$$

(2) $$b_{y}\;=\;\left(2\times \sigma \left(t_{y}\right)-0.5\right)\;+\;c_{y}$$

(3) $$b_{w}=p_{w}\times {\left(2\times \sigma \left(t_{w}\right)\right)}^{2}$$

(4) $$b_{h}=p_{h}\times {\left(2\times \sigma \left(t_{h}\right)\right)}^{2}$$

In this paper, the above Formula (1) and (2) are improved as:


(5) $$x\;=\;2.2\times \sigma \left(t_{x}\right)-0.6$$

(6) $$y\;=\;2.2\times \sigma \left(t_{y}\right)-0.6$$

(7) $$b_{x}\;=\;\{\begin{array}{c} -0.5\;,\;\;\;x<-0.5\;\\ x\;,\;\;\;\;\;\;\;\;other\;\;\\ 1.5\;,\;\;\;\;\;x>1.5\;\; \end{array}+\;c_{x}$$

(8) $$b_{y}\;=\;\{\begin{array}{c} -0.5\;,\;\;\;y<-0.5\;\\ y\;,\;\;\;\;\;\;\;\;other\;\;\;\\ 1.5\;,\;\;\;\;\;y>1.5\;\; \end{array}\;+\;c_{y}$$

where *b*_*x*_ , *b*_*y*_ , *b*_*w*_ , and *b*_*h*_ denote the locations of the predicted target centroid and height and width, respectively. σ is a Sigmoid function that aims to limit the predicted offset to between 0 and 1. *t*_*x*_ and *t*_*y*_ denote the offset of the x and y coordinates of the target center predicted by the network relative to the upper left corner of the grid, respectively, and *c*_*x*_ and *c*_*y*_ denote the x and y coordinates of the corresponding upper left corner of the grid. When the real target centroid falls at the center of the grid, the YOLOv5s model predicts the target by the grid where the centroid is located and the grid above and to the right of the grid where the centroid is located. When using the previous grid of the grid where the center point of the real target is located to predict the target, a value of *σ*(*t*_*y*_) of 1 is required according to the grid prediction offset formula, i.e., *t*_*y*_ needs to tend to positive infinity. When the target is predicted using the grid to the right of the grid where the center point of the real target is located, the value of *σ*(*t*_*x*_) needs to be 0 according to the grid prediction offset formula, i.e., *t*_*x*_ needs to tend to negative infinity. For these two cases, the original model formula is difficult to take these two values, so this paper improved the original formula by first increasing the range of values of the original formula to include all the required values, and then limiting the range of values so that it did not exceed the maximum value required for the grid prediction offset, and finally the formula obtained does not need to converge to infinity to take the required values.

#### 2.2.4 Improvement of the loss function

The loss of the YOLOv5s model consists of three components, including classification loss, confidence loss, and bounding box loss. Among them, BCE loss is used for classification loss and confidence loss, and GIoU loss is used for localization loss. The BCE loss and GIoU loss are calculated as follows:


(9) $$\text{BCE loss }=\;-\frac{1}{N}\times \sum_{n=1}^{N}\left[y_{n}\times logx_{n}+\left(1-y_{n}\right)\times \text{log}\left(1-x_{n}\right)\right]$$

Where *y*_*n*_ denotes the true category, which generally takes the value of 0 or 1, *x*_*n*_ denotes the prediction confidence or target probability obtained by the Sigmoid function, and N is the number of positive and negative samples.


(10) $$\text{GIoU loss }=\;1-IoU+\frac{A^{c}-u}{A^{c}}$$

Where IoU is the intersection ratio of the area of the real frame and the prediction frame, *A* ^*c*^ is the minimum outer rectangle of the real frame and the prediction frame, and u is the area of the concatenated set of the real frame and the prediction frame. The GIoU loss function solves the problem that the loss is zero when the prediction frame and the real frame do not overlap in the IoU loss function, but the GIoU loss function also has some problems, such as when the real frame and the prediction frame have the inclusion phenomenon The GIoU loss function degenerates into the IoU loss function when the real frame and the predicted frame intersect, and the slow convergence in the horizontal and vertical directions. The DIoU loss function solves the problems encountered by the GIoU loss function by introducing the overlap area and the distance of the centroids, and speeds up the convergence of the model by directly minimizing the distance between the two target frames.The Formula of the DIoU loss function is shown as follows:


(11) $$DIoU=IoU-\frac{\rho^{2}\left(b,b^{gt}\right)}{c^{2}}$$

(12) DIoU loss=1−DIoU

Where *ρ* ^2^(*b*,*b* ^*gt*^) is the square of the Euclidean distance from the centroid of the real frame to the centroid of the predicted frame, and c is the diagonal distance between the closed region of the two target frames. Although the performance of the DIoU loss function is higher than that of the GIoU loss function, the DIoU loss function also has some problems, for example, when the distance between the two target frames is constant, the longer the distance of c is, the smaller the value of the DIoU loss function is, which indicates that the DIoU loss function may achieve the purpose of reducing the loss function by enlarging the prediction frame, In order to solve this problem, this paper improved the DIoU loss function by adding the denominator of the original formula by the square of the Euclidean distance between the real frame and the upper left corner of the prediction frame to reduce the size of the change in the DIoU value when the edge length of the prediction frame changes, thus speeding up the convergence of the model. At the same time, this paper obtained the formula ${\left(\frac{w}{w^{gt}}-1\right)}^{2}+1$ based on parabolic reasoning, which introduces the ratio of the width of the prediction frame and the real frame. When the side lengths of the prediction frame and the real frame are the same, the ratio of $\frac{w}{w^{gt}}$ will be 1, while when the side lengths of the prediction frame and the real frame are not the same, the ratio of $\frac{w}{w^{gt}}$ will be greater than 1 or less than 1. Therefore, only when the prediction frame and the real frame The formula can solve the problem that the loss value decreases when the edge length of the prediction frame expands or shrinks. The improved DIoU loss function is given by


(13) $$DIoU=IoU-\frac{\rho^{2}\left(b,b^{gt}\right)}{\rho^{2}\left(l,l^{gt}\right)+c^{2}}\times \left({\left(\frac{w}{w^{gt}}-1\right)}^{2}+1\right)$$

**Figure 5:**
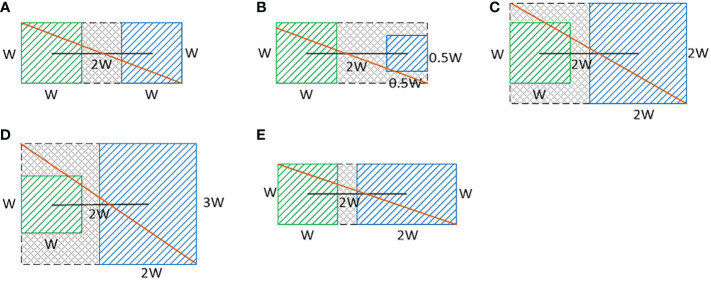
**(A–E)** Are the real box and prediction box matching diagram when the size of the prediction box is changed.

(14) DIoU loss=1−DIoU

where *ρ* ^2^(*l*,*l* ^*gt*^) denotes the square of the Euclidean distance between the upper left corner of the real frame and the prediction frame, and *w* ^*gt*^ and *w* denote the width of the real frame and the prediction frame, respectively. In this paper, five cases are exemplified to demonstrate the effectiveness of the improved loss function, as shown in **Figure 5**, in which the green box on the left represents the real frame, the blue box on the right represents the prediction frame, the black line is the center distance of the two target frames, and the orange line is the diagonal distance of the closed region of the two target frames. **Figure 5A** represents the best match between the real frame and the predicted frame, where the side lengths of both the real frame and the predicted frame are w and the distance between the two frame centroids is 2w. **Figures 5B–E** show the cases where the side lengths of the predicted frame change by keeping the side lengths of the real frame and the centroid distances of the two frames constant. Using the original DIoU loss function formula, we calculate the DIoU values of -0.4, -0.47, -0.25, -0.19, and -0.3 for the five figures in **Figure 5**. It can be seen that when the edge length of the prediction frame is enlarged, the DIoU value becomes larger, and thus the loss value decreases, so that the model converges in this direction and the prediction frame is wrongly enlarged, thus reducing the detection accuracy for small-scale samples. The DIoU values of the five images in **Figure 5** are -0.29, -0.37, -0.43, -0.33, and -0.54 using the improved DIoU loss function, which shows that the loss value increases when the edge length of the prediction frame is enlarged or reduced, so that the prediction frame trained by the model gradually matches the size of the real frame.

#### 2.2.5 Improvement of the YOLOv5s model

**Figure 6:**
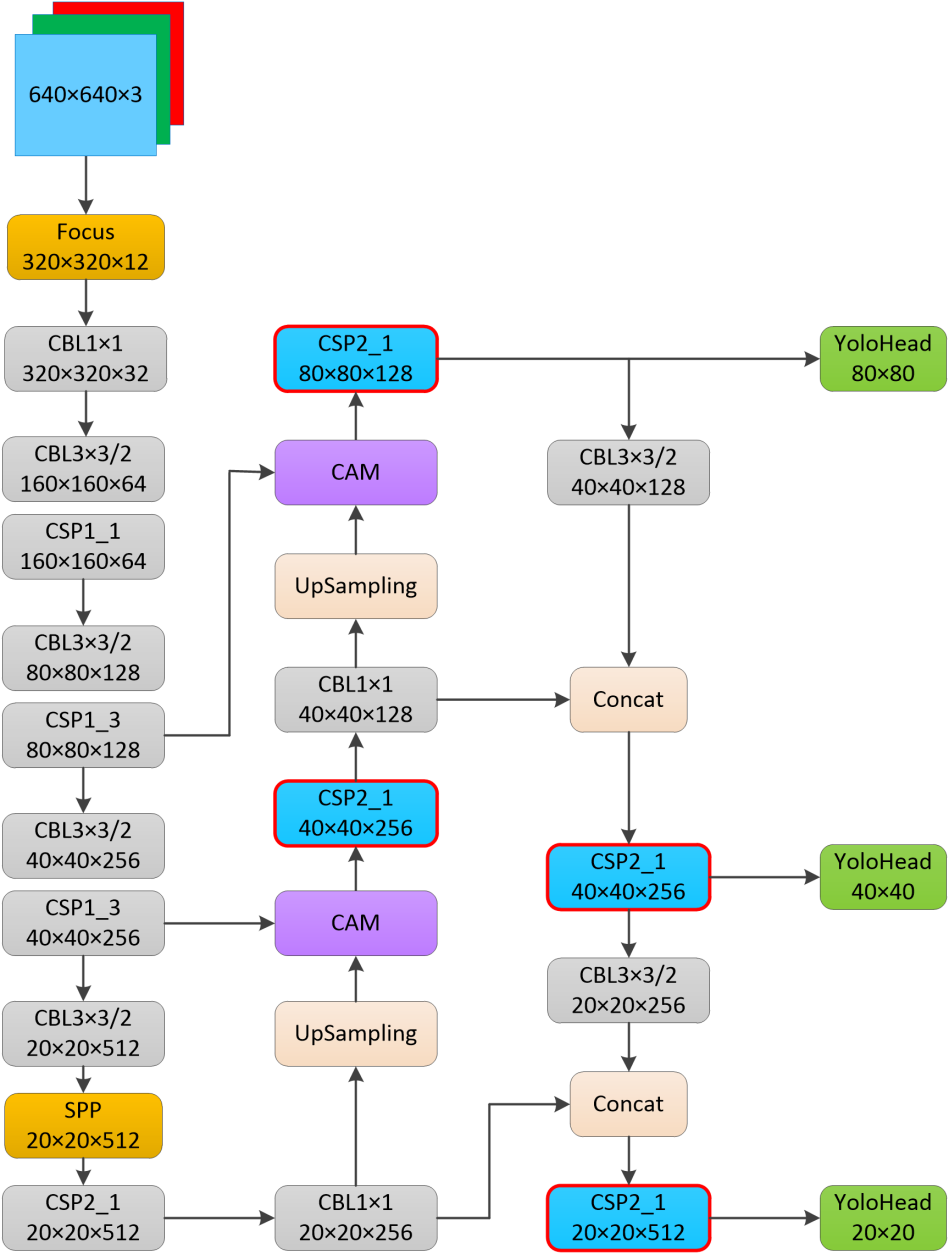
Improved YOLOv5s model.

There are four versions of the YOLOv5 model, s, m, l, and x. With the gradual expansion of depth and width, their parameters and calculation volume are also gradually increasing, and the number of parameters and computation of the four versions of the YOLOv5 model are shown in **Table 2**. YOLOv5s is one of the most lightweight models, and also has relatively low accuracy, while the various structures and formulas improved in this paper can improve the The YOLOv5s network model mainly consists of a backbone, a neck and a head. the backbone part includes the Focus module, the CSPdarknet53 structure and the SPP structure. Two CSP structures are designed in the YOLOv5s model, and different CSP structures are used for the backbone part and neck part. The improved YOLOv5s model in this paper is shown in **Figure 6**. The structural improvement was divided into two parts, one was to use the improved CSP structure instead of the original CSP structure in the neck of the YOLOv5s model, as shown in the red border in **Figure 6**. The improved model in this paper did not perform the splicing operation after the up-sampling operation, but used a CAM module, and the output of the shallow network of the backbone and the output of the up-sampling operation were used as the input of the CAM module respectively. The improvements in formulas and parameters were also divided into two parts, one was the improvement of the formula for predicting the coordinate offset of the target centroid and adding one more prediction grid to increase the number of positive samples, and the other was the improvement of the loss function formula. In addition to the above improvements, the pre-defined anchor values were also adjusted in this paper.

**Table 2:** Comparison of different versions of YOLOv5 model.

Model	Parameters (M)	Calculation volume (G)
YOLOv5s	7.08	8.22
YOLOv5m	21.08	25.25
YOLOv5l	46.67	57.18
YOLOv5x	87.29	108.74

The anchor values used in the original algorithm were obtained based on the COCO dataset, and the original preset anchor values were too large relative to the dataset used in this study. A suitable anchor value could improve the accuracy of model detection and speed up model convergence, so this study used the IOU value as an indicator to determine the distance between samples, with the following Formula:


(15) d(box,centroid) = 1−IOU(box,centroid)

The box is the target bounding box, centroid is the bounding box selected as the center in the clustering, and IOU is the intersection ratio between the target bounding box and the center box of the clustering. Larger IOU values and smaller distances indicate better anchor values are obtained. Nine sets of suitable anchor values were obtained by re-clustering the used dataset using the k-means algorithm, and the new anchor values were (13, 10), (13, 14), (29, 32), (42, 39), (47, 54), (61, 46), (74, 70), (140, 115), and (106, 365). Three sets of smaller anchor values were used to predict larger targets, three sets of medium anchor values were used to predict medium targets, and three sets of larger anchor values were used to predict smaller targets. All the above improvement parts together form the final network model in this paper.

This paper used the lightest of the four versions of the YOLOv5 model, and in order to achieve a lighter model, we compared the effect of using the lighter network MobileNetv3 as the backbone of the model on the experimental results based on the improved model. Also, there were many widely used loss functions in the field of target detection, and this paper compares several of them with the improved loss function in this paper to demonstrate the advantages of the improved loss function in this paper.

## 3 Experimental operation environment and model training

The experiment was performed on a windows workstation equipped with two Intel Xeon Silver 4210 10Core @ 2.20GHz CPUs with 128GB of RAM and NVIDIA RTX 2080ti with 11GB of video memory for the GPU. The experiment was also performed with Python 3.8, pytorch 2.6, and CUDA 10.0 training environments.

In this paper, the SGD optimizer was used in the model training process, and the momentum parameter used inside the optimizer was set to 0.937. Since this paper used the SGD optimizer, if the initial learning rate of the model was set too small, it would lead to too slow convergence, so the initial learning rate was set to 0.01, and in order to find the optimal solution faster in the later stage of the model training, the learning rate was adjusted in a stepwise manner. The size of the training image input was 640×640, the number of training epochs is 400, and the early stop mechanism was introduced in the training process, which stops the model when the loss value of the validation set does not drop more than 20 times. The early stop mechanism was introduced in the training process to stop the model when the loss value of the validation set does not drop more than 20 times, so as to avoid overfitting on the training dataset. In order to accelerate the model convergence, this paper used migration learning for training, and the batch size was 16.

## 4 Experimental results and comparative analysis

### 4.1 Model evaluation indicators

To obtain accurate model detection results, the trained models were evaluated using the evaluation metrics of mAP, precision (P), recall (R), average precision (AP), harmonic average F1 value (F1), number of network parameters, calculation volume and detection speed. The threshold value of IOU was set at 0.5, and the formulas for P, R, AP, F1 and mAP are shown in formula (16-20).


(16) $$P=\;\frac{TP}{TP+FP}$$

(17) $$P=\;\frac{TP}{TP+FN}$$

(18) $$AP=\int_{0}^{1}P\left(R\right)dR$$

(19) $$F1=\frac{2PR}{P+R}$$

(20) $$mAP=\frac{\int_{q=1}^{Q}AP\left(q\right)}{Q}$$

Where TP denotes the number of correctly detected diseases, FP denotes the number of incorrectly detected diseases, and FN denotes the number of undetected diseases. P denotes the proportion of all targets predicted by the model that are correctly predicted, and R denotes the proportion of all real targets that are correctly predicted by the model. For each disease, a P-R curve can be drawn based on the values of Precision and Precision, and AP represents the area under the P-R curve, and the closer the area is to 1, the better the performance. In general, Precision and Recall are negatively correlated, and Recall tends to be low when Precision is high, so in order to balance these two indicators, F1 value is proposed, which represents the weighted summed average of Precision and Recall. mAP is the average of multiple categories of AP, which is the most commonly used evaluation index in target detection, where mAP Q in the formula represents the number of categories in the data set. FPS is used to evaluate the speed of target detection, i.e., the number of images that can be processed per second; the larger the FPS, the faster the model detection speed.

### 4.2 Comparative experiments of each improvement part

**Figure 7:**
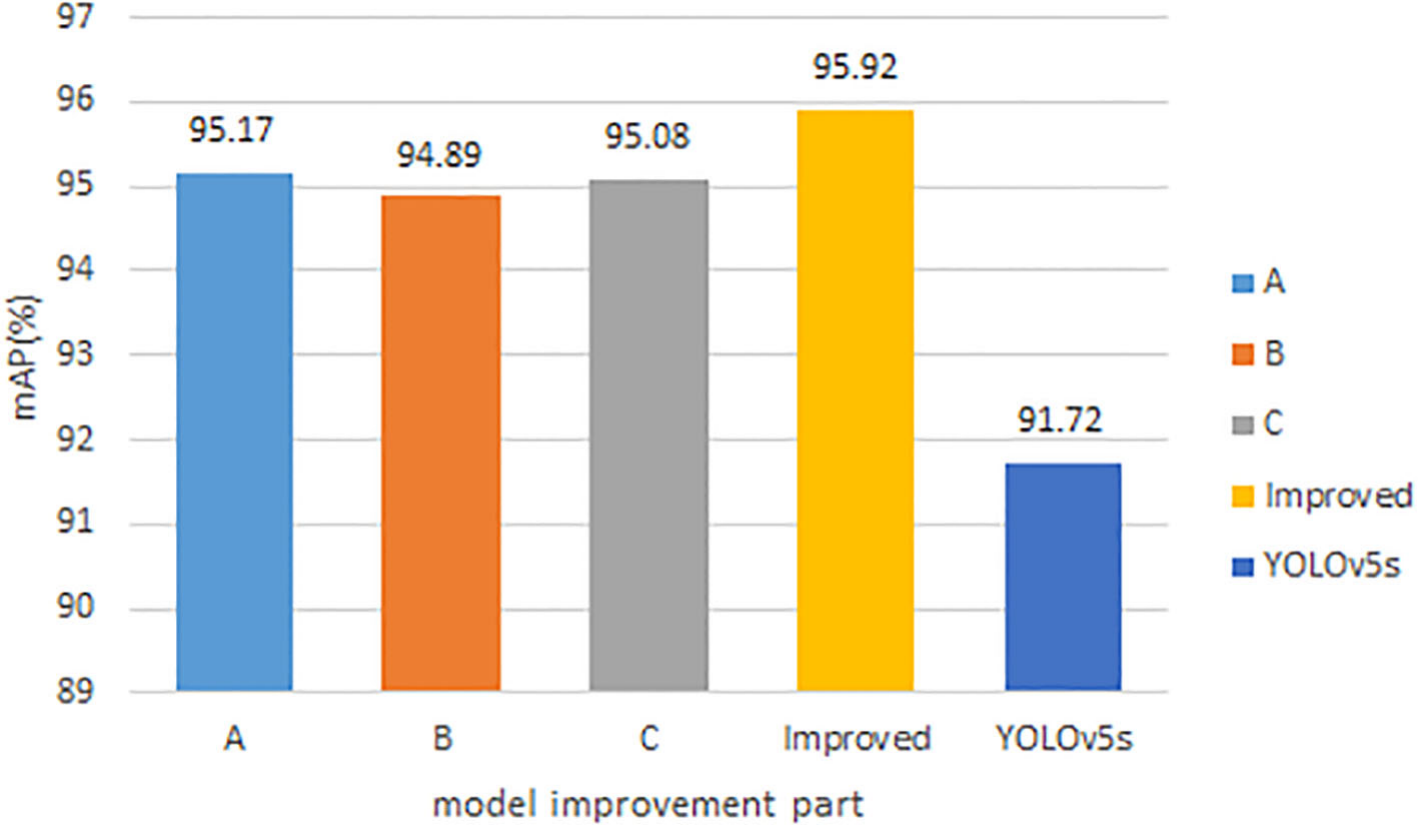
Comparison results of each improved part of the model.

The algorithm proposed in this paper was based on the improved YOLOv5s model. The improved aspects included the improvement of the neck CSP structure of the YOLOv5s model, a proposed CAM structure that enables better multi-scale fusion, the re-clustering of the anchor values, the improvement of the bounding box prediction method, and the improvement of the loss function. The expanded dataset was used as the training, validation, and testing samples, and the improved model in this paper was trained using the optimized nine sets of anchor values and the improved loss function. The results are shown in **Figure 7** for each improved part compared with the original model.

In the figure, A indicates the use of the improved CSP structure in the neck of the YOLOv5s model, B indicates the use of the proposed CAM structure in the YOLOv5s model, C indicates the optimization of the bounding box prediction method of the YOLOv5s model, and Improved is the final improved model that combines the above three improved parts. It can be found that the improved mAP values for each part of the original model in this paper are higher than the original model, and the final improved model is obtained by combining each improved part together, and the final improved model can get the highest mAP value.

There are eight disease types in the dataset used in this paper, and the results of comparing the AP values obtained from training in the improved model and the original model for each disease type are shown in **Table 3**. From the table, it can be seen that the final improved model has the best detection results for various diseases, and the AP values are higher than the original model.

**Table 3:** Comparative results of 8 disease types.

Type	Model	AP (%)	F1	Recall (%)	Precision (%)
Grape Black Measles	YOLOv5s	78.61	0.76	74.39	78.21
	improved-YOLOv5s	90.18	0.85	81.71	89.33
Grape Leaf Blight	YOLOv5s	96.30	0.91	88.18	93.27
	improved-YOLOv5s	98.65	0.92	88.18	97.00
Grape Black Rot	YOLOv5s	96.88	0.89	83.61	94.88
	improved-YOLOv5s	98.16	0.92	85.27	99.45
Peach Bacterial Spot	YOLOv5s	74.31	0.69	63.58	75.18
	improved-YOLOv5s	86.05	0.76	73.46	79.33
Potato Late Blight	YOLOv5s	98.34	0.97	96.09	97.79
	improved-YOLOv5s	99.31	0.97	97.83	96.98
Apple Black Rot	YOLOv5s	95.76	0.92	93.69	90.43
	improved-YOLOv5s	98.59	0.94	94.59	93.75
Apple Scab	YOLOv5s	95.03	0.89	86.27	92.15
	improved-YOLOv5s	96.85	0.93	89.46	97.33
Corn Northern Leaf Blight	YOLOv5s	98.56	0.92	85.19	98.92
	improved-YOLOv5s	99.60	0.96	92.59	100

### 4.3 Comparative experiments of different target detection models

In order to make the detection results more convincing, the improved model is compared with some commonly used target detection algorithms, such as Faster R-CNN, SSD, YOLOv3, YOLOv4, YOLOv4-tiny, and YOLOv5s, and the obtained experimental results are shown in **Table 4**. From the table, it can be seen that the number of parameters and computation of the YOLOv4-tiny model are substantially reduced compared with the YOLOv4 model, because the YOLOv4-tiny model deletes part of the structure of the YOLOv4 model, which makes the YOLOv4-tiny model structure more lightweight, but because the YOLOv4-tiny model structure is simpler and Faster R-CNN, as the main representative of the second-order target detection algorithm, uses RPN to generate candidate frames and projects the RPN-generated candidate frames onto the feature map to obtain the corresponding feature matrix, while the first-order target detection algorithm does not need to generate the candidate region stage and can directly generate The class probability and location information of the object, so the detection speed and computation of the Faster R-CNN algorithm is much higher compared to the first-order target detection algorithm. The improved model in this paper can achieve the highest accuracy with a small increase in the number of parameters and computation. The mAP value, F1 score, recall rate and accuracy of the improved model were improved by 4.58%, 5%, 4.78% and 4.5%, respectively, compared with the original model.

**Table 4:** Comprehensive performance comparison.

Model	mAP (%)	Params (M)	FLOPs (G)	F1	Recall (%)	Precision (%)
Faster R-CNN	65.45	28.35	473.28	0.62	69.41	57.04
SSD	84.83	24.68	30.80	0.60	48.75	93.95
YOLOv3	91.54	61.56	77.60	0.84	78.12	91.90
YOLOv4	87.21	63.98	70.78	0.78	70.42	89.79
YOLOv4-tiny	77.98	5.98	8.09	0.72	62.90	85.00
YOLOv5s	91.72	7.08	8.22	0.87	83.88	90.10
Improved-YOLOv5s	95.92	7.62	10.17	0.91	87.89	94.15

**Figure 8:**
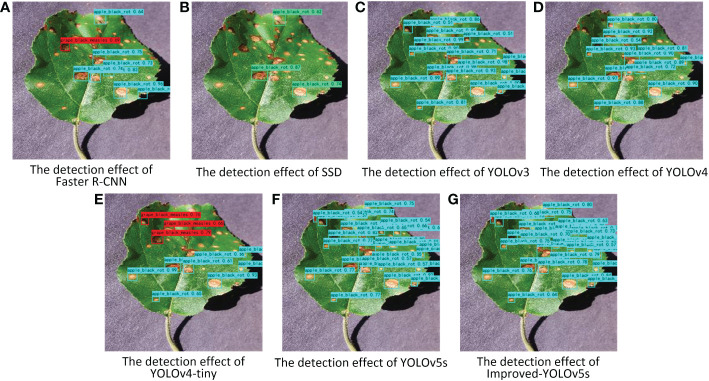
Pictures of the detection effect of apple black rot with different target detection models. **(A–G)** represent different target detection models.

**Figure 9:**
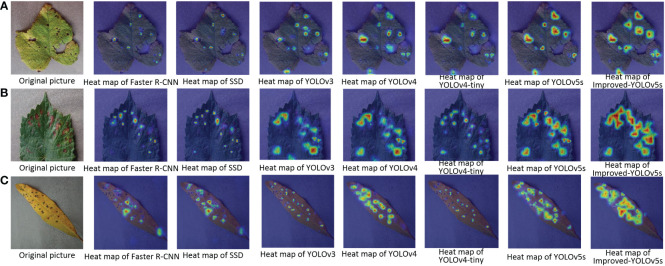
Pictures of the heat map detection of diseased leaves under different target detection models. **(A)** Grape Leaf Blight; **(B)** Grape Black Measles; **(C)** Peach Bacterial Spot.

In order to better illustrate the effectiveness of the improved model in crop disease detection, several disease detection images were selected, as shown in **Figure 8**. From the figure, it can be seen that the improved model in this paper can correctly detect the disease locations, and also effectively avoid the problems of missed and false detection for small and multiple targets. Meanwhile, the results of the heat map visualization of the three diseases in the dataset are shown in **Figure 9**, which shows that the improved model in this paper can focus on more disease areas. In summary, the improved YOLOv5s model proposed in this paper has the best detection effect.

### 4.4 Comparative experiments of different backbone network models

MobileNetv3 network was a lightweight convolutional neural network based on MobileNetv1 and MobileNetv2, which could significantly reduce the number of parameters and computation of the model compared with other classical networks in the direction of image classification. In order to compare the effect of using a lighter network as the backbone of the model on the experimental results, this paper compares the results after replacing the backbone of the improved model from CSPDarknet53 to MobileNetv3, as shown in **Table 5**. Except for replacing the backbone network with MobileNetv3, the rest of the model structures were consistent with the improved model proposed in this paper. Improved-MobileNetv3 was the backbone network of the improved model proposed in this paper replaced with MobileNetv3, and Improved-YOLOv5s was the improved model proposed in this paper, as can be seen from the table. Improved-MobileNetv3 model has a significant decrease in the number of parameters and computation, which is 10.45% and 20.19% lower than the original model. mAP value of the Improved-MobileNetv3 model is 2.92% higher than the original model, but it is lower compared with the backbone network which is CSPDarknet53. YOLOv5s-MobileNetv3 was the replacement of the backbone network of the original model in this paper with MobileNetv3. From the table, we can see that the YOLOv5s-MobileNetv3 model has the lowest number of parameters and computation, as well as the fastest detection speed, but also the lowest mAP value of 83.52%.

**Table 5:** Comparison results of different backbone models.

Model	mAP (%)	Params (M)	FLOPs (G)	F1	Recall (%)	Precision (%)	FPS
YOLOv5s	91.72	7.08	8.22	0.87	83.88	90.10	41.12
YOLOv5s-MobileNetv3	83.52	5.80	4.61	0.56	47.68	93.78	49.19
Improved-MobileNetv3	94.40	6.34	6.56	0.89	85.51	92.90	43.76
Improved-YOLOv5s	95.92	7.62	10.17	0.91	87.89	94.15	40.01

**Figure 10:**
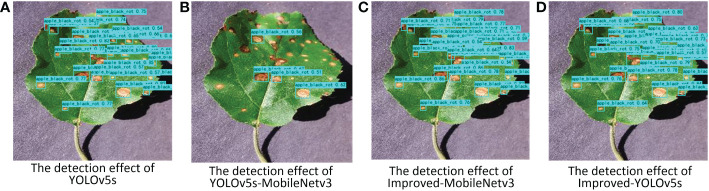
Pictures of the detection effect of apple black rot under different backbone networks. **(A)** YOLOv5s model; **(B)** YOLOv5s-MobileNetv3 model; **(C)** Improved-MobileNetv3 model; **(D)** Improved-YOLOv5s model.

**Figure 11:**
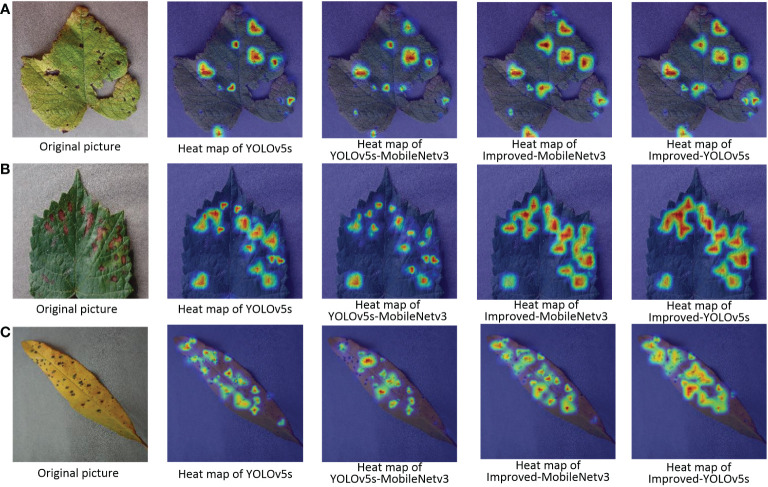
Pictures of the heat map detection of diseased leaves under different backbone networks. **(A)** Grape Leaf Blight; **(B)** Grape Black Measles; **(C)** Peach Bacterial Spot.

The pictures of the detection effect of this paper for apple black rot under different backbone networks are shown in **Figure 10**, from which it can be seen that the YOLOv5s-MobileNetv3 model can detect the least number of diseases correctly although it has the lowest number of parameters, while the Improved-MobileNetv3 model can detect most of the disease locations, and the detection effect is second only to the Improved-YOLOv5s model. The results of the heat map display for the three disease types for different backbone network models are also shown in **Figure 11**, which shows that both Improved-MobileNetv3 and Improved-YOLOv5s models were able to focus on more disease areas, and the color degree for the disease areas was also deeper, with Improved-YOLOv5s model being able to The Improved-YOLOv5s model can achieve the best detection effect. In summary, among the improved models, the model obtained using CSPDarknet53 as the backbone network has the most comprehensive performance, while using MobileNetv3 network as the backbone network can reduce the number of parameters, but at the same time, the detection effect will be reduced.

### 4.5 Comparative experiments of different loss function

**Figure 12:**
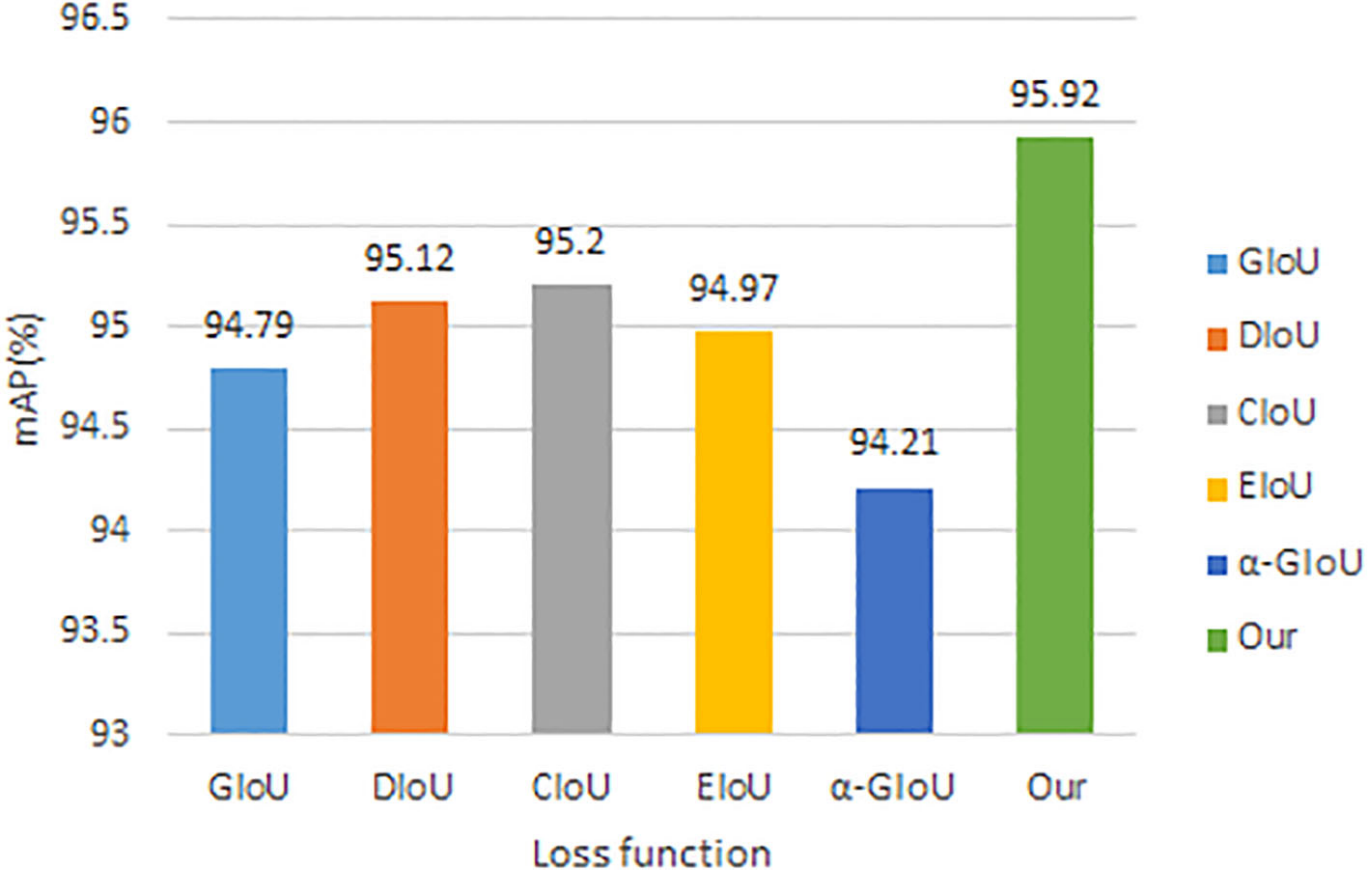
Comparison results of different loss function.

In this paper, the performance of the improved loss function was verified by using several loss functions commonly used in current target detection in the improved model to compare with the improved loss function, and the obtained experimental results are shown in **Figure 12**. The results in the figure show that the highest mAP value can be achieved by using the improved loss function in this paper, and the higher accuracy is obtained by introducing the ratio of the Euclidean distance between the upper left corner of the real frame and the prediction frame and the width of the two target frames based on the DIoU loss function to suppress the enlargement or reduction of the prediction frame errors.

## 5 Discussion

In this paper, we used some common data enhancement methods and mosaic data enhancement methods to process the training data. In this paper, we found that if we simply used common data enhancement methods or mosaic data enhancement methods, we couldn’t obtain the best results. The data enhancement method could improve the generalization ability of the model and improve the robustness of the model, and the mosaic data enhancement method could greatly enrich the background of the detected objects, but the method will cause the training images to be out of the real distribution of the natural images, so the mixture of the normal data enhancement method at a certain ratio could make the model get better detection results. This paper improves the CSP structure used in the neck of the original YOLOv5s model. The original CSP structure divided the input into two branches, and then spliced the outputs of the two branches together, which could enhance the fusion ability of the network features. The experimental results showed that the mAP value of the model was only slightly improved because the number of small targets in the dataset used in this paper was the majority. For the design of the CAM structure, the ability to extract both global and local information from the feature map was considered. In the initial design, only one input from the deep network was considered, and the input was allowed to perform global average pooling and global maximum pooling operations respectively, but the results were not satisfactory. The later designed CAM structure then contains two parts of the input, the shallow network had a low abstraction level and contains more detailed information, while the deep network contained more semantic information. The CAM structure integrates the feature information of the shallow network and the deep network well and enhanced the global information extraction ability of the network. The Tanh function converges faster than the Sigmoid function, and this paper was more efficient in predicting the The Tanh function was used instead of the Sigmoid function in the formula involved in predicting the coordinate offset of the target centroid, while adjusting the value domain of the Tanh function, but it did not achieve better results. The reason was that the Tanh function was more sensitive to the value of function value change between (-1,1), which tends to level off earlier than the Sigmoid function, and the Sigmoid function converges more smoothly than the gradient of the Tanh function. So finally, this paper improved the formula of Sigmoid function, by limiting the range of values of the formula to get the value needed in this paper. In the process of improving the loss function, we tested many formulas, some of which could solve the problem of the prediction frame being wrongly enlarged, but would lead to the problem of the prediction frame shrinking and the loss value decreasing, and the model converging in the wrong direction. There were also many other scholars who have improved the YOLOv5 model and applied it to different datasets, for example, some scholars had detected the presence of powdery mildew and anthracnose in rubber trees based on the improved YOLOv5 model, and the results showed that the improved model has an average accuracy of 70%. We trained this improved model on the dataset we used and obtained an average accuracy of 92.58%, and our improved model gave better results compared to our own model. Other scholars detected tomato disease images based on the improved YOLOv5 model and could get 94.10% mAP. Currently, most scholars was detecting on single or few plant diseases, while our improved model can detect on eight plant diseases and achieve 95.92% mAP. In comparison, our improved model not only obtained higher average accuracy but also was able to detect more types of diseases. In the future, we would continue to research lighter and more effective models to detect more types of crop diseases, and we would study the deployment of lightweight models to robots for real-time detection.

## 6 Conclusion

An improved YOLOv5s model was proposed for detecting several common crop diseases with small, dense and overlapping crop disease targets. First, eight diseases in the PlantVillage dataset were manually labeled to obtain the dataset for training. Second, the Ghost module and the inverted residual block were incorporated into the CSP structure of the YOLOv5s neck to construct a lightweight CSP structure, and the improved CSP structure was able to extract more feature information. Meanwhile, a CAM module was constructed in which the inverse residual block and ESPA module was fused, and feature fusion of different scale feature maps was also achieved by extracting global and local information from different layer networks. Then, the number of positive samples for model training was increased by adding one more prediction grid, while the formula of grid prediction offset was modified so that the offset of the center point of the prediction frame could be taken more easily to the value under the particular point. The problem of slow convergence of the model training and the problem of obtaining smaller loss values by wrongly enlarging and shrinking the prediction frame during the model training was solved by improving the formula of the loss function. Finally, the K-menas algorithm was used to re-cluster the dataset used in this paper to obtain the appropriate anchor values to go for training. The experimental results showed that the improved YOLOv5s model had stronger global information extraction capability, better accuracy and robustness for crop disease detection, and better identification of smaller disease targets, while the improved model had less number of parameters and computational effort also lays the foundation for deploying the model to embedded or mobile devices.

## Data availability statement

The original contributions presented in the study are included in the article/supplementary material. Further inquiries can be directed to the corresponding author.

## Author contributions

YZ and YY designed the research and performed the experiments and data analysis, and wrote the manuscript. XX revised the manuscript, CS collected material data. All authors contributed to the article and approved the submitted version.
